# The optimal grouting area and cable distribution design for guaranteeing roadway stability while considering fluid mechanical effects via the CMOEAD algorithm

**DOI:** 10.1038/s41598-025-85611-0

**Published:** 2025-01-07

**Authors:** Peng Li, Yinghai Guo, Yinghao Cheng, Jiaming Zhang

**Affiliations:** 1 School of Resources and Earth Sciences, China University of Mining and Technology, Xuzhou, China; 2 The First Hydrogeological Team of China Coal Geology Administration, Handan, China; 3 Key Laboratory of Coalbed Methane Resources and Accumulation Process of Ministry of Education, Xuzhou, China

**Keywords:** Water inrush, Fluid‒mechanical coupling effect, Cable, Optimization, CMOEAD (constrained multi-objective evolution algorithm based on decomposition) algorithm, Mechanical engineering, Natural hazards

## Abstract

Water inrush in roadways frequently occurs in coal mines when the rock mass is enriched with underground water. To avoid underground water flow into the roadway and guarantee the stability of the roadway, grouting and cables are commonly used to prevent water inrush and guarantee the stability of the roadway. In this work, FLAC3D (fast lagrangian analysis of continua 3 dimension) numerical simulation software was used, and the fluid‒mechanical coupling effects were considered. In combination with the CMOEAD (constrained multi-objective evolution algorithm based on decomposition) optimization method, the optimal grouting area and cable distribution were determined: the center point of the ellipse (grouting area) is (0.01, 1.59), the long axis length is 4.73 m, the short axis length is 4.60 m, and the inclination angle of the ellipse is 53.15°. The cable length is 6.51 m, the total number of cables is 11. The grouting area and cable distribution design from numerical simulation results were applied to engineering practice, the degree of water inrush was markedly reduced, and the displacement of the roadway was within control, indicating that the proposed method is workable and reliable.

## Introduction

Coal is the main energy source in China; 95% of energy, 80% of industrial materials and 70% of agricultural materials are produced using coal^1,2^. With the extensive exploitation of coal in simple geology areas, coal sources in these areas have gradually decreased. Hence, coal mining in complex geology areas is becoming increasingly important. Due to complex geology conditions, some mine disasters, such as water disasters and roadway deformation, have a great influence on safe coal mining production.

There are many fractures and voids in a rock mass, and these fractures, fissures and voids provide good channels for water seepage. Moreover, the water pressure may result in further development of crack propagation and crack coalescence in the rock mass, increasing the fissure and fracture areas and ultimately increasing the risk of water inrush^3^ and roadway deformation. The coupling effect between underground water and rock mass mechanics makes the interaction mechanism complicated. To investigate the coupling effects of underground water and rock mass mechanics, numerical simulation is a good choice. Wang et al.^4^ investigated hydraulic fracturing behavior considering the effects of in situ stress, fracture density and fracture length; in numerical tests, through analysis of numerical simulation results, the degree of damage to and degree of activation of fractures decreased with increasing number of natural fractures. This study provides an experimental basis for the coupling effects between the number of fractures and rock fracture. To simulate the hydraulic fracturing process via numerical simulation, the numerical manifold method was used, and a numerical manifold-based fluid‒solid coupling model was proposed^5^. To verify the validity of the proposed method, several examples were considered by comparing the experimental tests and numerical simulation results, indicating that the proposed method is reliable and workable. Sun et al.^6^ proposed a three-dimensional resolved numerical framework to simulate rock‒fluid interactions, compressible smoothed particle hydrodynamics were applied to depict hydrodynamic behavior, and the discrete element method was used to simulate the mechanical behavior of grain materials. An analysis of the numerical simulation results shows that the solid‒fluid coupling interaction can be reflected comprehensively by the proposed method and that the method is reliable. Many other researchers have also studied the rock‒fluid coupling effect^7–10^, and these studies provide a numerical basis for waterproof coal mining.

A better understanding of the coupling effects of underground water and rock is conducive for water prevention and guarantees roadway stability. In engineering practice, different techniques have been applied for water prevention and to guarantee roadway stability. Gu et al.^11^ proposed a dynamic stability criterion from the perspectives of energy and stress. On the basis of this criterion, a corresponding water prevention method was proposed. In this method, the degree of damage to the coal segment in surrounding coal should receive enough attention, and their study provides valuable insights into water prevention design and guarantees roadway stability. Liu et al.^12^ analyzed the geological conditions and aquifer structural characteristics of the Wutongzhuang coal mine, and several in situ tests were conducted. On the basis of the test results, numerical simulations were performed, and the optimal water prevention and optimal support design of roadways was proposed. In this study, numerical simulations and in situ tests were combined to determine the water prevention and optimal support design of roadways, which is meaningful. Owing to the merits of numerical simulations, many other researchers have developed water prevention techniques and optimal support designs for roadways via numerical simulations^13–19^.

Numerical simulation is an effective way to obtain the optimal water prevention and cable support design for roadways. However, through an analysis of the above references, it can be found that water prevention and optimal roadway support design are dependent mainly on the researcher’s experience, which indicates that the determination process is not objective enough and that the water prevention design and optimal roadway design may vary among different researchers, in the other words, the specific water prevention and roadway support techniques would be varied when the geotechnical condition is changed, which indicated the application of most water prevention and roadway support method is limited, and it can not be applied to engineering practice directly. To avoid the subjectivity of the determining process, in this paper, the CMOEAD (Constrained Multi-Objective Evolution Algorithm based on Decomposition) algorithm and FLAC3D (fast lagrangian analysis of continua 3 dimension) numerical simulation were combined via the Python script language. To verify the validity of the proposed method, a numerical simulation example was given. By using the proposed method, the optimal grouting area and cable support were obtained, the optimal design was applied to engineering practice, and the stability of the roadway was guaranteed, which indicated that the proposed method is reliable and workable.

## Determining the optimal grouting area and cable support design of roadways via the CMOEAD algorithm

Grouting and cables are effective ways to prevent water from flowing into the roadway and to guarantee the stability of the roadway. In this work, grouting was implemented by changing the mechanical parameters and porosity coefficient. To guarantee the stability of the roadway, a cable unit was used to support the roadway.

### Fluid‒mechanical coupling in FLAC3D

In this work, FLAC3D numerical simulation software was used, and deformation and fluid diffusion occurred simultaneously as water flooded the roadway^19^. The formulation of coupled deformation‒fluid diffusion processes in FLAC3D is performed within the framework of quasistatic Biot theory and can be applied to problems involving single-phase Darcy flow in a porous medium. Various types of fluids, including water and oil, can be represented in FLAC3D.

For the fluid, the characteristic length can be expressed as:

1 $$L_{c} = \frac{volume \, of \, flow \, domain}{{surface \, area \, of \, flow \, domain}}$$

The fluid diffusivity can be denoted as:

2 $$c = \frac{k}{{1/M + \alpha^{2} /\alpha_{1} }}$$

where $k$ is the mobility coefficient, $M$ is the Biot modulus, $\alpha$ is the Biot coefficient, $\alpha_{1} = K + 4/3G$,$K$ is the drained bulk modulus, and $G$ is the shear modulus of the porous material.

The Biot coefficient takes into account the grain compressibility of the porous material. If $\alpha$ is equal to unity, the grains are considered incompressible, and the Biot modulus $M$ is equal to $K_{f} /n$, where $K_{f}$ is the fluid bulk modulus and $n$ is the porosity; then, the fluid diffusivity becomes:

3 $$c = \frac{k}{{1/K_{f} + 1/\alpha_{1} }}$$

_The fluid transport is described by Darcy’s law:_

4 $$q_{i} = - k_{il} \hat{k}(s)[p - \rho_{f} x_{j} g_{j} ]_{,l}$$

where $q_{i}$ is the specific discharge vector, $p$ is the fluid pore pressure, $k$ is the tensor of absolute mobility coefficients of the medium, $\hat{k}(s)$ is the relative mobility coefficient, which is a function of fluid saturation, and $\rho_{f}$ is the fluid density.

_For small deformation, the fluid mass balance can be expressed as:_

5 $$- q_{i,i} + q_{v} = \frac{\partial \zeta }{{\partial t}}$$

where $q_{v}$ represents the volumetric fluid source intensity and where $\zeta$ represents the variation in the fluid content of the fluid volume per unit volume of porous material due to diffusive fluid mass transport.

The balance of momentum has the form:

6 $$\sigma_{ij,j} + \rho g_{i} = \rho \frac{{dv_{i} }}{dt}$$

where $\rho = \rho_{d} + ns\rho_{w}$ is the bulk density, $\rho_{d}$ and $\rho_{w}$ are the densities of the dry matrix and the fluid, respectively, $n$ is the porosity, and $s$ is the saturation.

Changes in fluid content are related to changes in pore pressure $p$, saturation $s$ and mechanical volumetric strain $\varepsilon$.

7 $$\frac{1}{M}\frac{\partial p}{{\partial t}} + \frac{n}{s}\frac{\partial s}{{\partial t}} = \frac{1}{s}\frac{\partial \zeta }{{\partial t}} - \alpha \frac{\partial \varepsilon }{{\partial t}}$$

where $M$ is the Biot modulus, $n$ is the porosity, and $\alpha$ is the Biot coefficient.

### Mechanical behavior of the cable unit

In FLAC3D, the cable model is suitable for explaining the axial reinforcing mechanism. The axial stiffness $K$ is determined on the basis of the reinforcement cross-sectional area $A$, Young’s modulus $E$, and cable length $L$ via the following relation:

8 $$K = \frac{AE}{L}$$

**Fig. 1:**
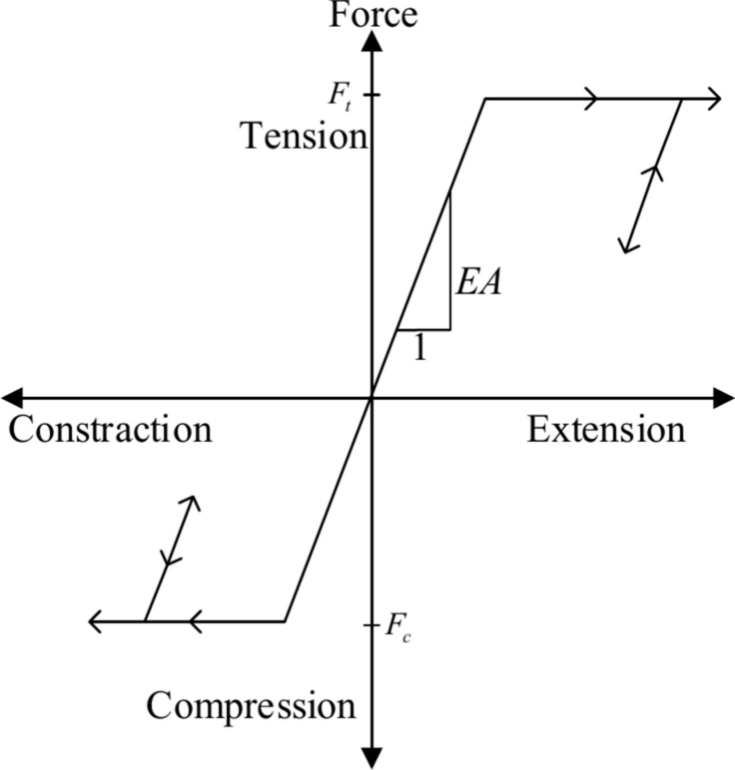
Axis mechanical limits of cables^20^.

The tensile yield strength $F_{t}$ and compressive yield strength are assigned to the anchor cable element; when the strength is greater than these limits, the cable fails, which is shown in (Fig. 1).

**Fig. 2:**
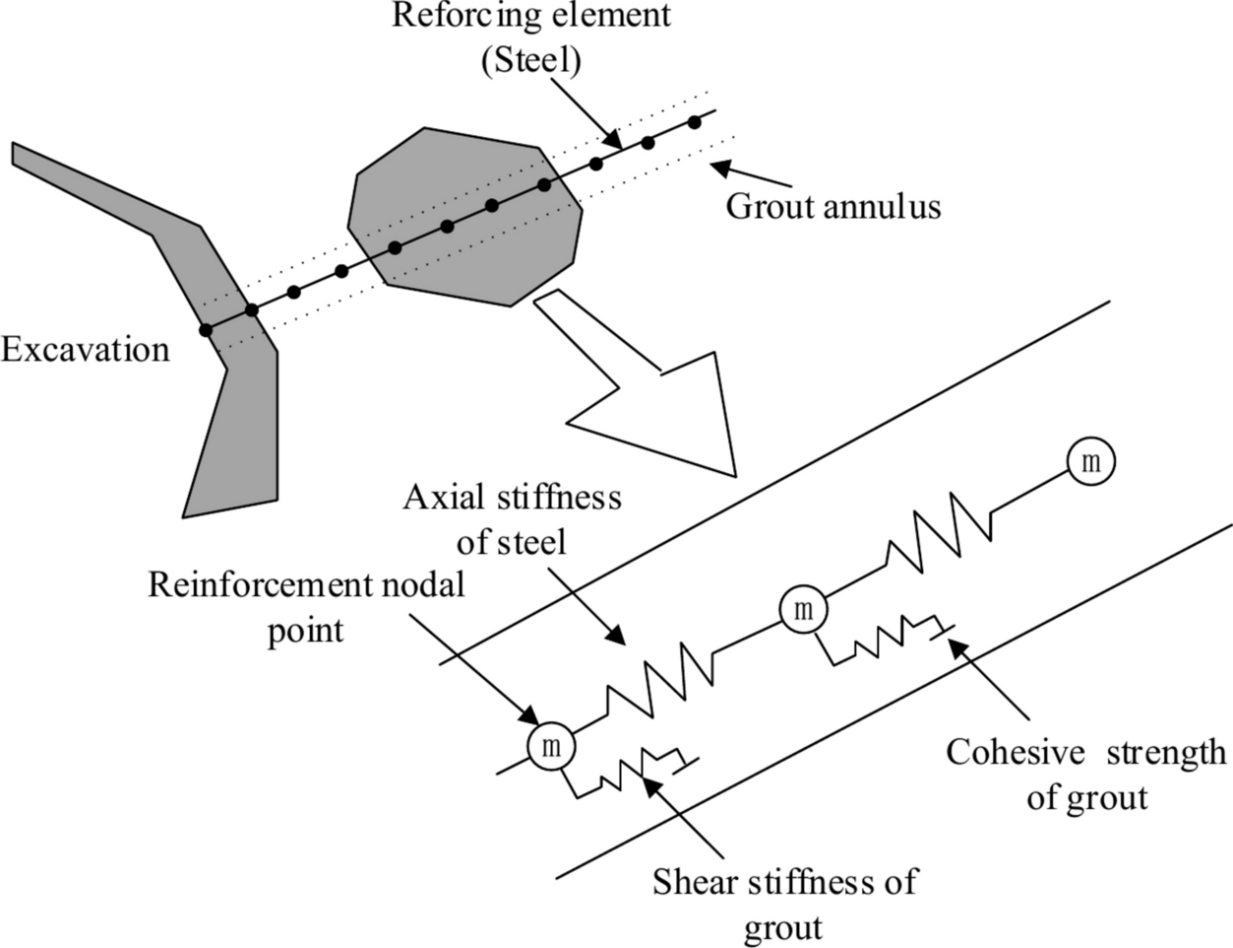
Axial mechanical representation of a cable^20^.

To estimate the axial forces of the reinforcement, displacements are calculated on the basis of nodal points along the reinforcement direction, as shown in (Fig. 2). Axial displacements are computed by integrating the nodal accelerations via the out-of-balance axial force and a mass lumped at each node.

**Fig. 3:**
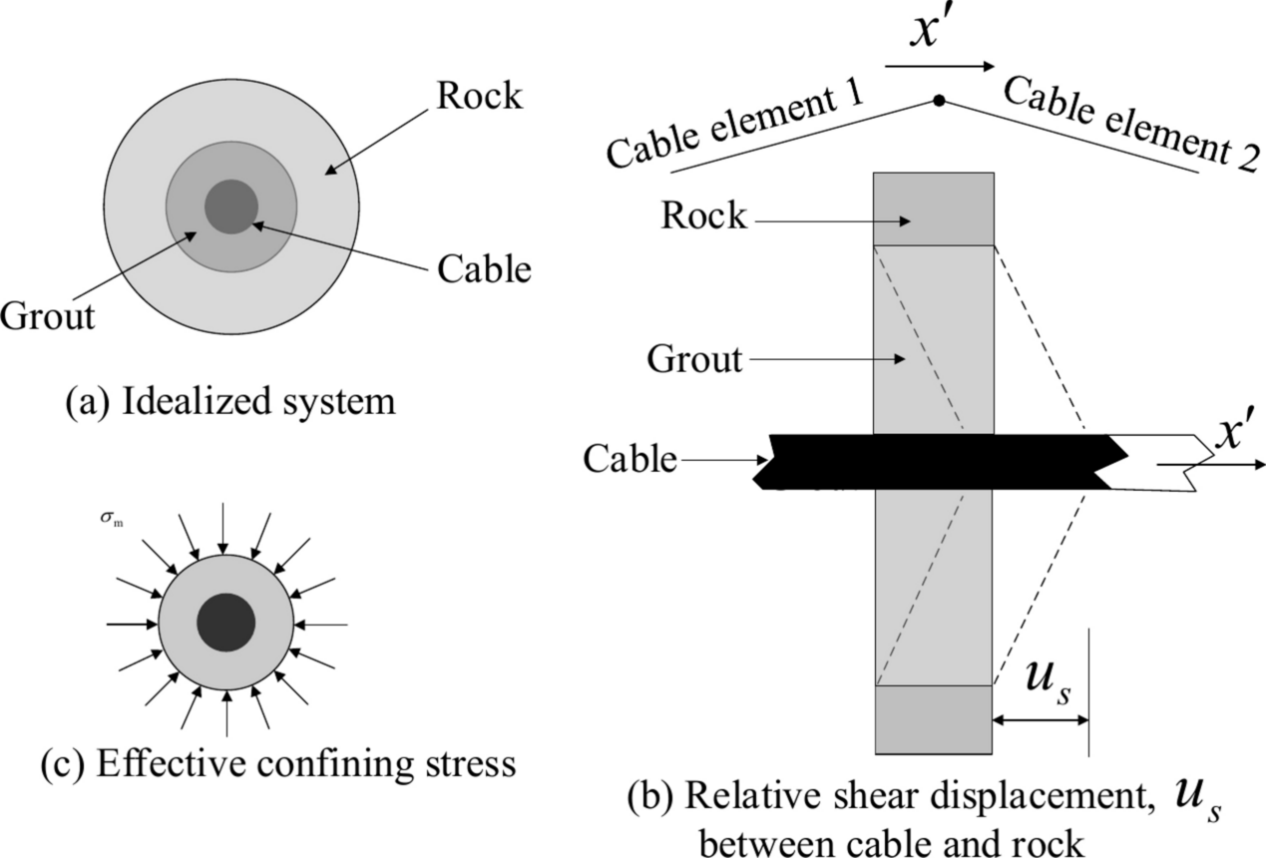
Grout-cable system^20^.

The shear behavior of the cable‒rock interface is naturally cohesive and frictional, as described in Fig. 3a, and it is depicted by using a spring‒slider system located at the nodal points along the cable axis (Fig. 3b,c). The relationship between the rock mass and cable is described numerically by the grout shear stiffness $k_{g}$, grout cohesive strength $c_{g}$, grout friction angle $\varphi_{g}$, grout exposed perimeter $p_{g}$, and effective confining stress $\sigma_{{\text{m}}}$. Notably, the grout properties associated with each cable are averaged at the cable nodes.

**Fig. 4:**
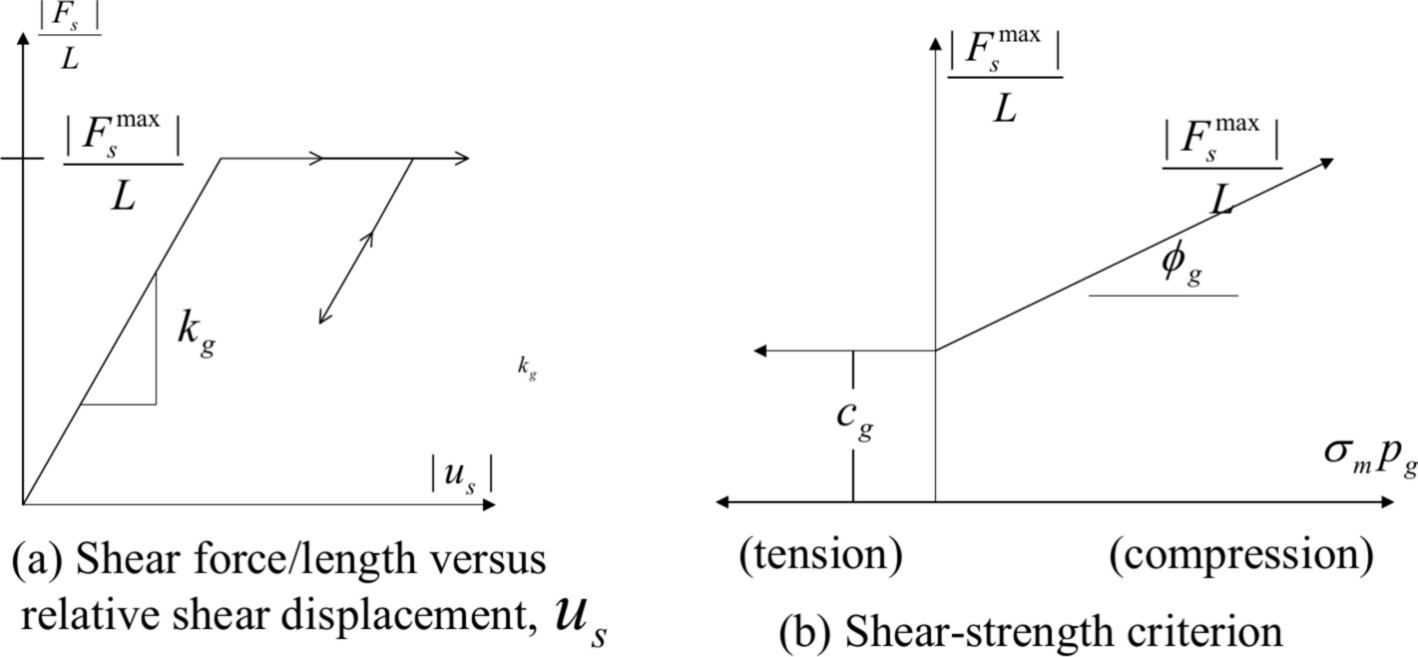
Grout and cable material mechanical behavior^20^.

The mechanical behavior between the rock mass and grouting can be expressed as shown in (Fig. 4).

### CMOEAD optimization algorithm

For roadway support and water prevention, two methods were used: grouting and cables. In engineering practice, overgrouting and long cables can guarantee the safety of roadways and prevent water inrush into roadways. However, this approach is not economical. When the grouting area and cable length are insufficient, the roadway can collapse, or underground water can flow into the roadway, resulting in death and economic loss. Therefore, determining the optimal grouting area, number of cables and length of cables can guarantee the safety of the roadway and save much of the expense.

The grouting area and cable distribution are two different variables, and the objective problem involves the displacement of the roadway and water inrush of the roadway. This is a typical multiple objective optimization problem, and the grouting area, number of cables and length of the cables should be determined.

Traditionally, different kinds of grouting area and cable design would be given, and the optimal method would be selected from the limited design, as a matter of fact, the optimal grouting area and cable design may not included in the limited proposed method. To solve the multiple optimization problem, in this paper, the CMOEAD (constrained multi-objective evolution algorithm based on decomposition)^21^ was used to determine the minimum grouting area and minimum cable length. For the CMOEAD algorithm, the multiple objective problem was transformed into a single objective problem, and the PBI (penalty-based boundary intersection) mechanism was adopted to guarantee the convergence and diversity of the algorithm.

For the CMOEAD algorithm, in the boundary intersection based on the penalty method, the distance between the objective vector and reference point is used to find the optimal solution:

9 $$g^{bip} (x|\lambda ,z*) = d_{1} + \theta d_{2}$$

where

10 $$d_{1} = \frac{{||(z* - F(x)^{T} \lambda )||}}{||\lambda ||}$$

11 $$d_{2} = ||F(x) - (z* - d_{1} \lambda )||$$

In the equations, $g^{bip}$ is the double standard function, which is used for estimating the quality of the solution; $x$ is the present solution; $\lambda$ is the reference direction, which is used for the search process; $z*$ is the ideal point, which is the best estimate for $F(x)$; $\theta$ is the weight of $d_{2}$; and $d_{1}$ is the projection distance of $x$ along the $\lambda$ direction.

The PBI method can be used to solve the multiple-objective problem effectively and can guide the search process. Moreover, the diversity of the population is guaranteed, and the convergence speed is fast.

The procedure of the CMOEAD algorithm is as follows:

- Step 1: Transform the multiple-objective problem into a single-objective problem by assigning weights to each problem.
- Step 2: Initialize the population of the solution; the initial population is treated as a structure solution, and the number of populations is *N*.
- Step 3: Transform the minimum value as the ideal point, take the ideal point as the coordinate, and normalize all the solutions on the basis of the ideal point.
- Step 4: Generate the new population on the basis of two parent generations (the process is similar to that of the genetic algorithm).
- Step 5: Determine the fitness of the solution via PBI techniques.
- Step 6: Terminate the iteration until the termination condition is satisfied.

**Fig. 5:**
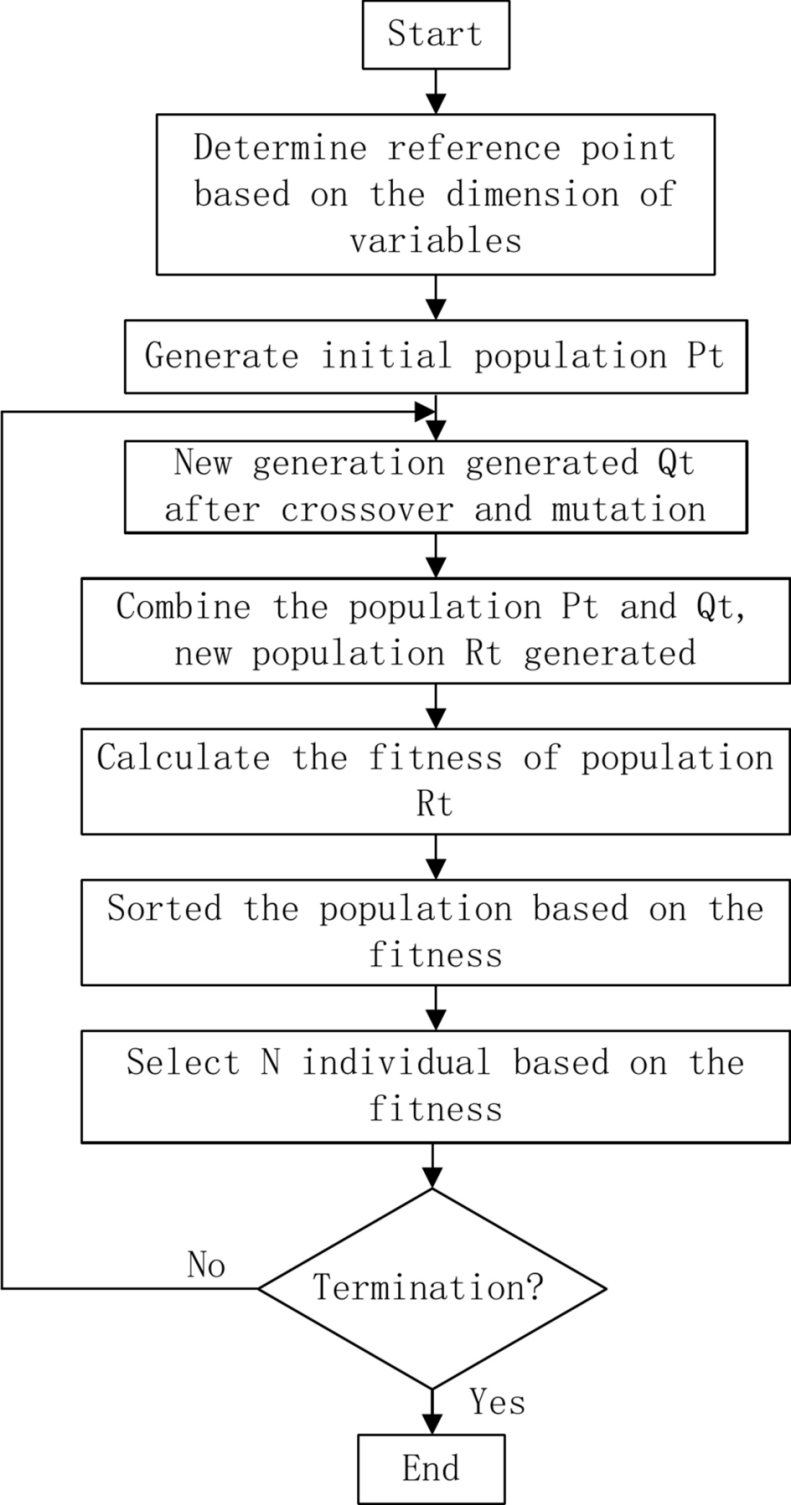
Flow chart of the CMOEAD algorithm.

For convenience in implementing the CMOEAD algorithm, the flow chat is shown in (Fig. 5).

### Determining the optimal grouting area and cable distribution via the CMOEAD algorithm

In this work, two objective problems are considered: the grouting area and the cable distribution. In engineering practice, both the cable length and the grouting area should be small enough. Moreover, the stability and safety of the roadway should be guaranteed. For convenience of numerical simulation, the stability and safety of the roadway are described by three constraint conditions: the relative movement of the roof and floor should be less than or equal to 0.3 m, the displacement of the two sides should be less than or equal to 0.3 m, and the water flow into the roadway should be less than or equal to 0.2 m^3^/h^22^. These constraint conditions can be changed on the basis of the standards of engineering practice.

**Fig. 6:**
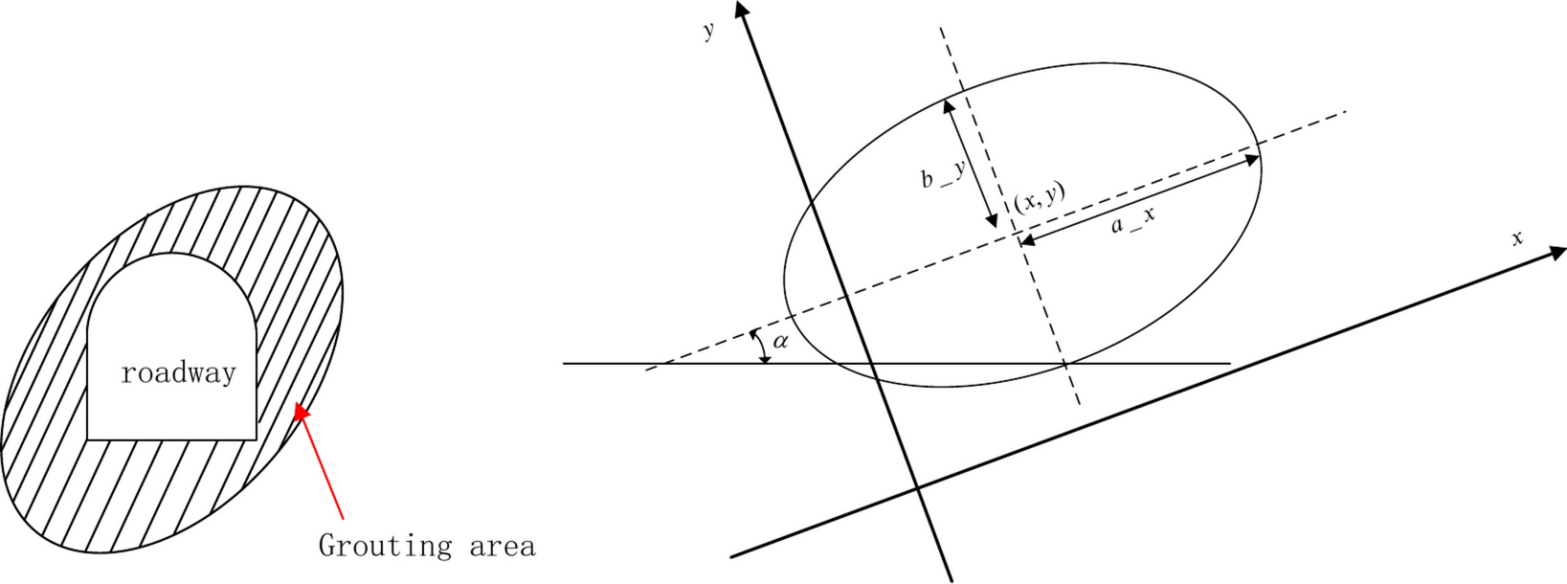
Geometric parameters of the grouting area.

To avoid water flow into the roadway, the grouting technique was adopted. In this work, the grouting area was ellipse-shaped, and four geometric parameters controlled the shape of the ellipse (Fig. 6): the center of the ellipse (*x*,* y*), the long axis length a_x, the short axis length *b*_*y*, and the inclination angle of the ellipse $\alpha$. The grouting area was the area where the area of the cross-sectional area of the roadway was subtracted.

In engineering practice, the grouting area should be small enough, and the water flow into the roadway should be less than 0.2 m^3^/h. Furthermore, to guarantee the stability of the roadway, cables were also used.

**Fig. 7:**
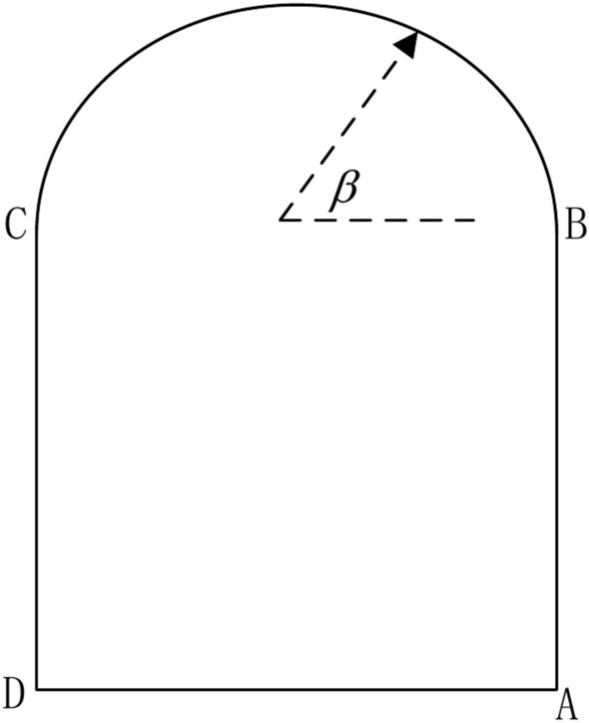
Cable inclination angle.

For the cable, the starting point of the cable is fixed on the surface of the roadway, and the other points of the cable can be expressed via the following equation:

12 $$\left\{ \begin{gathered} x_{2} = x_{1} + L\cos \alpha_{2} \hfill \\ y_{2} = y_{1} + L\sin \alpha_{2} \hfill \\ \end{gathered} \right.$$

where (*x*_1_, *y*_1_) are the start points of a cable, and this point is on the surface of the roadway. *L* is the length of the cable. Notably, the length of the cable is the same, which is convenient for numerical simulation. $\alpha$^2^ is the inclination angle of a cable. A cable has a corresponding inclination angle, and different cables have different inclination angles (Fig. 7).

During the numerical simulation, the cable inclination angle ranges were different when the start point of the cable was located. As illustrated in Fig. 7, when the start point is located from point A to point B, the inclination angle of the cable is from −90 to 90°, and when the start point of a cable is within the range of arc BC, the inclination angle should be −(90−$\beta$) to 90 + $\beta$. When the start point of the cable is from point C to point D, the inclination angle ranges from 90 to 270°. Hence, the inclination angle ranges are determined once the start point of a cable is determined.

Moreover, the end point (*x*_2_,*y*_2_) of a cable should be in the rock mass, and cross-connections among cables should be avoided. Finally, the number of cables should also be determined. In summary, the variables of the cables included the number of cables, length of the cables, cable start points, and cable angles. The objective function is that the total length of the cables should be small enough, the relative movement of the roof and floor should be less than or equal to 0.3 m, and the displacement of the two sides should be less than or equal to 0.3 m.

In summary, the variables of water prevention and roadway support can be expressed as the center of the ellipse (*x*, *y*), long axis length *a_x*, short axis length *b_y*, inclination angle of the ellipse $\alpha$, number of cables *N*, start points for each cable, cable length *L* (all cable lengths are the same), and inclination angle of each cable. However, the objective of the problem is that the grouting area and the total length of the cable should be small enough. The constraint conditions are that the relative movement of the roof and floor should be less than or equal to 0.3 m and that the displacement of two sides should be less than or equal to 0.3 m. The water flow into the roadway should be less than 0.2 m^3^/h.

The process of obtaining the optimal grouting area and cable distribution via the CMOEAD algorithm is described below.

Step 1: Initialize the parameters of the CMOEAD algorithm and the ranges of the variables (ellipse (*x*, *y*), long axis length *a_x*, short axis length *b_y*, inclination angle of ellipse $\alpha$, number of cables *N*, start points for each cable, cable length *L* (all cable lengths are the same), and inclination angle of each cable.

Step 2: On the basis of the ranges of the variables and hyperparameters of the CMOEAD algorithm, the initial solutions of the numerical simulation are generated.

Step 3: Based on the grouting area parameters (ellipse parameters) and cable parameters (cable number, cable length and cable location), the FLAC3D command flow was generated, and numerical simulations were conducted. On the basis of the numerical simulation results (relative movement of the roof and floor, water flow into the roadway), if the roof and floor are less than or equal to 0.3 m, two-sided displacement should be less than or equal to 0.3 m, and the water flow into the roadway should be less than 0.2 m^3^/h. The objective value can be calculated on the basis of the CMOEAD algorithm, or the objective value should be infinity.

Step 4: Adjust the variables on the basis of the CMOEAD algorithm.

Step 5: If the termination condition is satisfied, the numerical simulation is terminated, and the process continues to step 6. If the numerical simulation is not terminated, go to step 2.

Step 6: End.

To implement the process, the Python script language was used, the numerical simulation process was completely controlled by the CMOEAD algorithm and Python scripts until the termination condition was satisfied, the numerical simulation was terminated, and the optimal grouting area and the optimal cable design were determined. To verify the validity of the proposed method, a numerical simulation example was given.

### Application in engineering practice

**Fig. 8:**
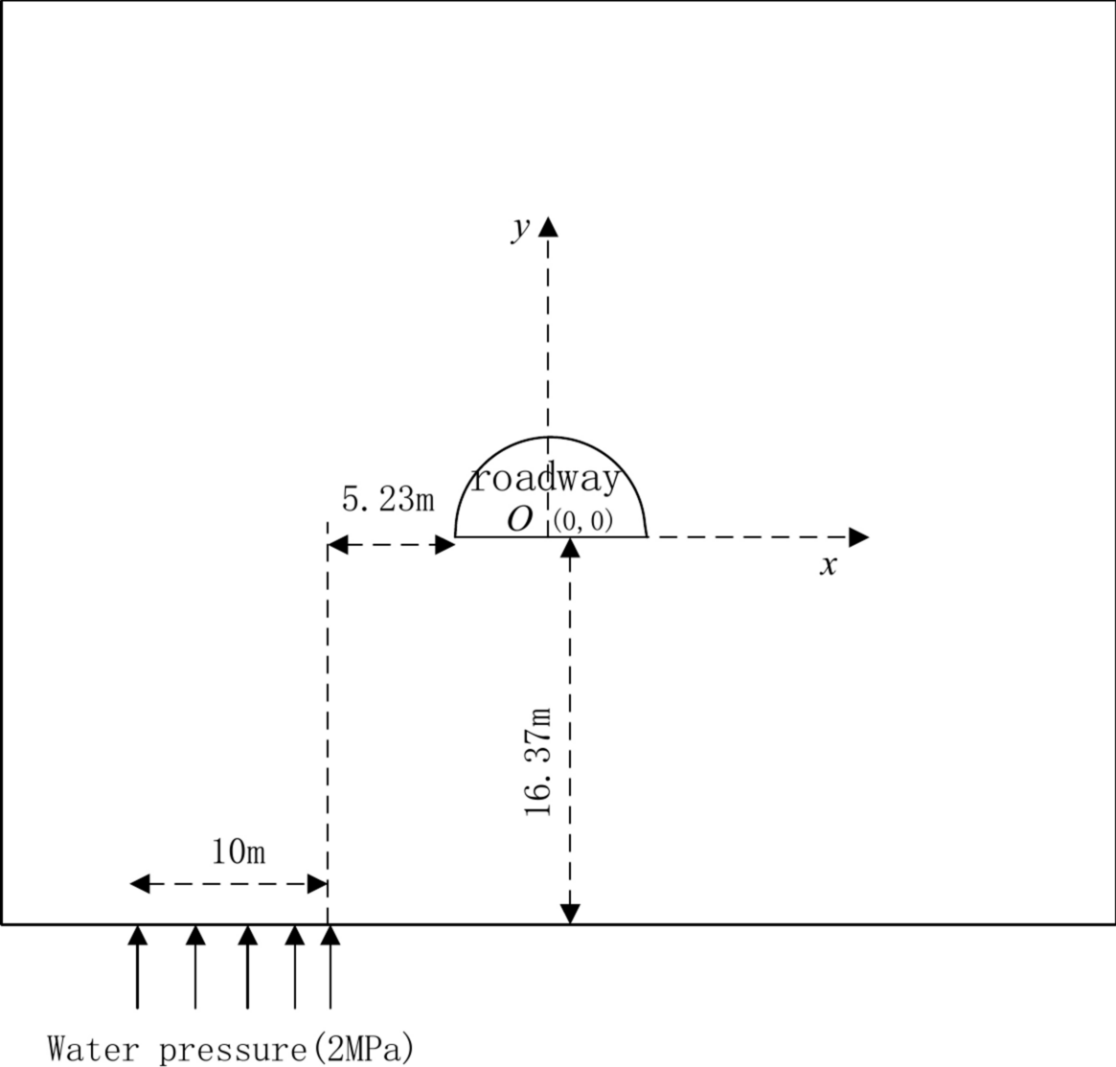
Numerical simulation model.

To validate the proposed method, numerical simulations were conducted. The roadway of the PanEr Coal Mine of Huanan was selected as an example. The roadway height was less than 600 m, the height of the roadway was 3.3 m, the width of the roadway was 5.54 m, the speed of water flow into the roadway was 113 m^3^/h, the relative displacement of the roof and floor was 1.5 m, and the relative displacement of the two sides was 0.65 m. The underground water location is shown in (Fig. 8). Water flowed into roadways, and the large displacement of roadways resulted in a large threat to daily production.

To determine the optimal cable location and grouting area, the FLAC3D numerical simulation model was constructed, and the center coordinate of the roadway floor was the location of point (0, 0), the mechanical parameters of rock mass and grouting area was listed in (Table1).

**Table 1:** Mechanical parameters of the rock mass and grouting area.

	Density(kg/m^3^)	Bulk modulus(GPa)	Shear modulus(GPa)	Cohesion(MPa)	Friction angle(^o^)	Tension(MPa)
Rock mass	2500	3	9e9	1.3	9	0.5
Grouting area	2600	3.5	10e9	5	31	2

The permeability coefficient of the rock mass is 8.7e-11, whereas the permeability coefficient of the grouting area is 5.0e-13.

Furthermore, the mechanical parameters of the cables are listed in (Table 2).

**Table 2:** Mechanical parameters of the cables.

Young’s modulus(Pa)	200e9	Yield tension(Pa)	1320e6	Grout friction angle(^o^)	30
Poisson’s ratio	0.2	Grout stiffness(Pa)	1.12e7		
Section area(m^2^)	379e-6	Grout cohesion(Pa)	1.75e5		

By using the CMOEAD algorithm, the optimal grouting area and cable design are obtained, and the geometry of the grouting ellipse area parameters are as follows: the center point of the ellipse is (0.01, 1.59), the long axis length is 4.73 m, the short axis length is 4.60 m, and the inclination angle of the ellipse is 53.15°. The cable length is 6.51 m, the total number of cables is 11, and the start and end points of the 11 cables are listed in (Table 3).

**Table 3:** Start points and end points of the 11 cables.

Series number	Start point	End point
1	(2.77, 0)	(9.27,0)
2	(2.63, 1.02)	(8.81, 3.03)
3	(2.24,1.94)	(7.50,5.76)
4	(1.63,2.67)	(5.45,7.93)
5	(0.85,3.14)	(2.87,9.32)
6	(0.01,3.29)	(0.01,9.80)
7	(−0.85,3.13)	(−2.85,9.32)
8	(−1.63,2.67)	(−5.44,7.93)
9	(−2.24,1.93)	(−7.49,5.77)
10	(−2.63,1.02)	(−8.81,3.04)
11	(−2.76,0.01)	(−9.26,0.01)

**Fig. 9:**
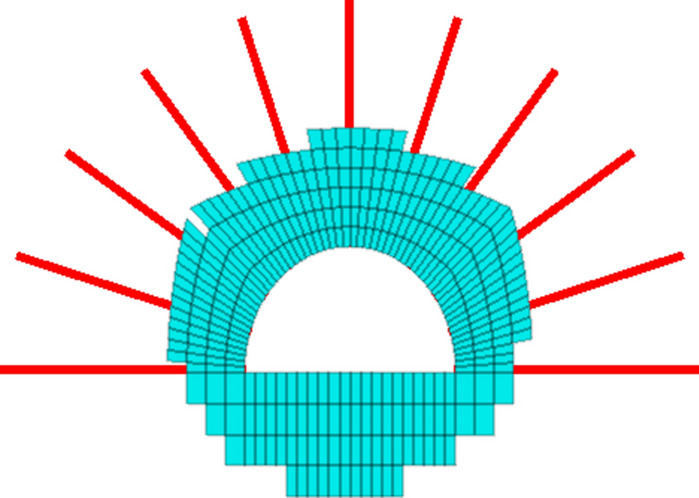
Grouting area and cable distribution.

The grouting area and the cable distribution are shown in (Fig. 9).

**Fig. 10:**
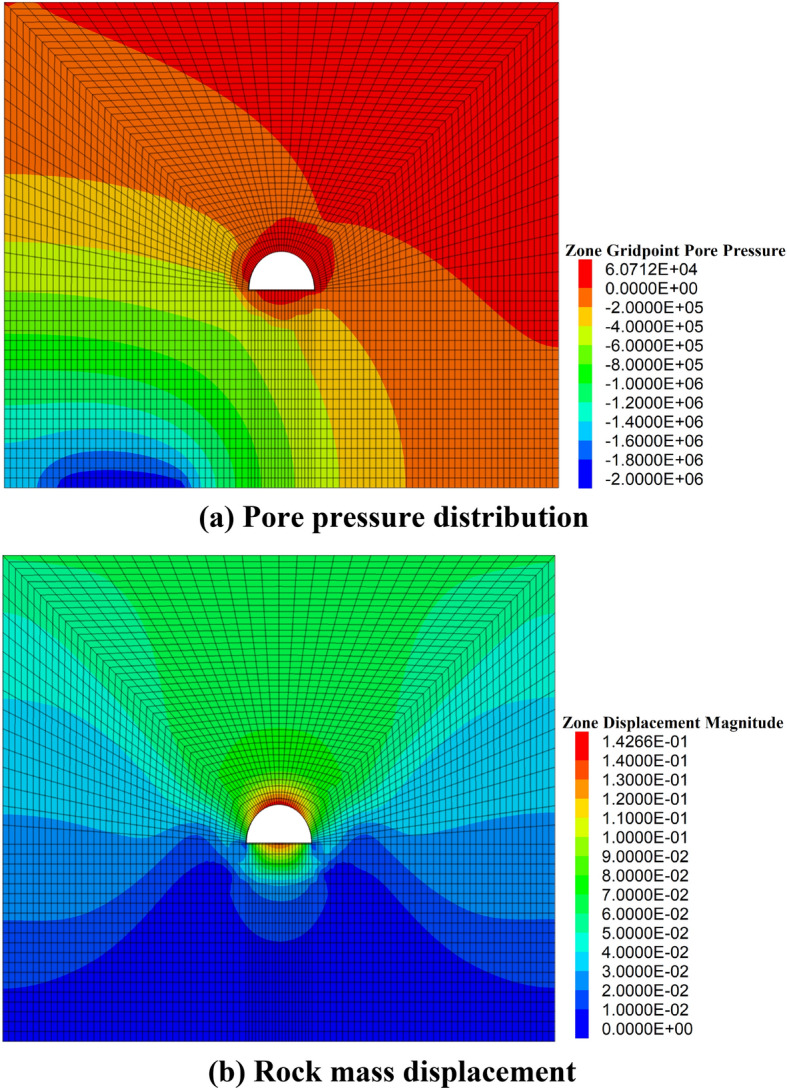
Numerical simulation results. (**a**) Pore pressure distribution, (**b**) Rock mass displacement.

By using the grouting area and cable design, the pore pressure distribution around the roadway is shown in Fig. 10a, and the speed of water flow into the roadway is 0.18 m^3^/h, which is less than 0.2 m^3^/h.

The rock mass of the roadway is shown in (Fig. 10b). The relative displacement of the roof and floor is 0.26 m, and the relative displacement of the two sides is 0.18 m, which is less than the standard displacement.

To verify the validity of the numerical simulation results, the grouting area and cable distribution design are applied to engineering practice, the water flow into the roadway is 0.19 m^3^/h, the relative displacement roof and flow is 0.23 m, and the relative displacement of the two sides is 0.19 m, which are quite similar to the numerical simulation results. Both the numerical simulation results and the engineering tests indicate that the proposed method is reliable and can be applied in engineering practice.

## Discussion

Underground water inrush into roadways and the deformation of roadways commonly occur in the mining process. Underground water inrush into roadways is a major threat for safe production. In addition, underground water can soften the rock mass, and its mechanical parameters can be reduced, resulting in large deformation of the roadway or even collapse.

Grouting is an effective way to prevent underground water, and cables can reduce the deformation of roadways. In engineering practice, to guarantee the safety and stability of roadways, excessive grouting and cables commonly appear in engineering, which is not economical. However, insufficient grouting and cables can cause instability in roadways, which is dangerous. Through the analysis above, obtaining the minimum grouting area and minimum cable length is critically important, and the stability of the roadway should be guaranteed. To solve this problem, the CMOEAD algorithm and FLAC3D numerical simulation software were combined, and the optimal geometry parameters and cable parameters were adjusted on the basis of the CMOEAD algorithm. To implement the process, the corresponding Python script language was used. To verify the validity of the proposed method, a numerical simulation example was given, the optimal grouting area parameters and cable design parameters were obtained, and these parameters were applied to engineering practice. Both the numerical simulation results and in situ tests indicated that the proposed method is reliable and workable and can be applied to engineering practice. Meanwhile, through analysis of numerical simulation process, it can be found that the determining optimal grouting area and cable design is independent on the researcher’s experience, once the edge of deep roadway was determined, the optimal support design can be determined, these proposed method can be applied to in situ with more complex geological condition.

In this work, only two methods to prevent water inrush and support roadways (grouting and cables) and are used; in real-world scenarios, other supporting techniques would be used. Moreover, the proposed method should be verified with more examples.

## Conclusions

Water flow into roadways and roadway deformation are great threats for coal mining. Grouting is effective for water inrush prevention, and cables are a good choice for reducing roadway deformation; however, determining the optimal grouting area and cable distribution is remains still a challenge. In this work, the CMOEAD algorithm and FLAC3D numerical simulation were combined to obtain the optimal grouting area and cable distribution via the Python script language. The main conclusions of this paper can be summarized as follows.

1. To determine the optimal grouting area and cable design distribution, the CMOEAD algorithm and FLAC3D numerical simulation software were combined via the Python script language, and the grouting area and cable distribution were adjusted via the CMOEAD algorithm.
2. To validate the proposed method, the roadway in the PanEr coal mine was taken as an example. In the numerical simulation, the minimum grouting area and cable distribution were used, and the numerical simulation results were applied to engineering practice, indicating that the proposed method is reliable and workable. Meanwhile, through analysis of numerical simulation process, it can be found that the determining optimal grouting area and cable design is independent on the researcher’s experience, once the edge of deep roadway was determined, the optimal support design can be determined, these proposed method can be applied to in situ with more complex geological condition or with other support techniques. However, in our manuscript, only one example was given, hence, more engineering practice would be used to verify the validity of the proposed technique, it would be our next task.

## Data Availability

The datasets used and/or analysed during the current study available from the corresponding author on reasonable request.
